# Characterization of a monoclonal antibody with specificity for holo-transcobalamin

**DOI:** 10.1186/1743-7075-3-3

**Published:** 2006-01-04

**Authors:** Lars Orning, Anne Rian, Andrew Campbell, Jeff Brady, Sergey N Fedosov, Birgit Bramlage, Keith Thompson, Edward V Quadros

**Affiliations:** 1 Axis-Shield AS, POB 206 Økern, N-0510 Oslo, Norway; 2 Protein Chemistry Laboratory, Department of Molecular and Structural Biology, University of Aarhus, Science Park, Gustav Wieds Vej 10, 8000 Aarhus; 3 Institute of Immunology, Rikshospitalet University Hospital, University of Oslo, N-0027 Oslo, Norway; 4 Division of Hematology/Oncology, State University of New York, Health Science Center, Brooklyn, NY 11203, USA; 5 Alnylam Europe AG, Fritz-Hornschuch-Str. 9, 95326 Kulmbach, Germany

## Abstract

**Background:**

Holotranscobalamin, cobalamin-saturated transcobalamin, is the minor fraction of circulating cobalamin (vitamin B12), which is available for cellular uptake and hence is physiologically relevant. Currently, no method allows simple, direct quantification of holotranscobalamin. We now report on the identification and characterization of a monoclonal antibody with a unique specificity for holotranscobalamin.

**Methods:**

The specificity and affinity of the monoclonal antibodies were determined using surface plasmon resonance and recombinant transcobalamin as well as by immobilizing the antibodies on magnetic microspheres and using native transcobalamin in serum. The epitope of the holotranscobalamin specific antibody was identified using phage display and comparison to a de novo generated three-dimensional model of transcobalamin using the program Rosetta. A direct assay for holotrnscobalamin in the ELISA format was developed using the specific antibody and compared to the commercial assay HoloTC RIA.

**Results:**

An antibody exhibiting >100-fold specificity for holotranscobalamin over apotranscobalamin was identified. The affinity but not the specificity varied inversely with ionic strength and pH, indicating importance of electrostatic interactions. The epitope was discontinuous and epitope mapping of the antibody by phage display identified two similar motifs with no direct sequence similarity to transcobalamin. A comparison of the motifs with a de novo generated three-dimensional model of transcobalamin identified two structures in the N-terminal part of transcobalamin that resembled the motif. Using this antibody an ELISA based prototype assay was developed and compared to the only available commercial assay for measuring holotranscobalamin, HoloTC RIA.

**Conclusion:**

The identified antibody possesses a unique specificity for holotranscobalamin and can be used to develop a direct assay for the quantification of holotranscobalamin.

## Background

The clinical consequences of vitamin B12 (cobalamin; Cbl) deficiency include megaloblastic anemia and progressive neurologic disease of the central and peripheral nervous systems. The elderly population is especially prone to this deficiency because of reduced dietary intake and impaired absorption of the vitamin [1]. Elevated homocysteine (HCY) and methylmalonate (MMA) in blood are a direct consequence of B12 deficiency and these metabolites have been implicated in vascular damage and neurotoxicity, respectively [2,3].

Cbl in serum is bound to two proteins, transcobalamin (TC) and haptocorrin (HC). Most of the Cbl in blood is bound to HC, a purported scavenger protein and a transporter for mobilization of hepatic stores of Cbl, though, the exact function of HC remains unknown [4]. Approximately 10–30% of the TC in the blood is saturated with Cbl (holotranscobalamin, holoTC). TC carries the vitamin from its site of absorption to the tissues [5] and delivers the vitamin to cells via a specific receptor with high affinity for holoTC [6].

Measurement of Total Cbl in serum has been for a long time the gold standard for evaluating the Cbl status of patients. However, it is now evident that total serum Cbl is a rather poor indicator of Cbl status because elevated HCY and MMA, (indicators of intracellular Cbl deficiency) can coexist with normal serum Cbl levels [7]. It has been suggested that direct determination of holoTC may provide a better indicator of Cbl status because it reflects daily Cbl absorbed and the fraction that is available for cellular uptake [8,9]. Although this hypothesis was originally suggested some 17 years ago [10], reliable and sensitive methods for measurement of holoTC became available only recently [11,12]. These methods are manual and thus not suitable for handling the large number of samples required in the clinical laboratory. In this report, we describe the identification and characterization of a mouse monoclonal antibody (mAb) with high specificity for human holoTC and its usefulness in an ELISA based prototype assay for holoTC in serum.

## Methods

Encapsulated magnetic microspheres (EM1 100/40; mean diameter, 0.86 μm) coated covalently with goat anti-mouse IgG (H+L) antibody were from Merck-Eurolab SAS (France). ^57^Co labeled cyanoCbl was from MP Biomedicals (UK). Unlabeled cyanoCbl was from Sigma (Norway). Rabbit anti-mouse Fc-γ used for immobilization of murine mAbs on a SPR sensor chip was from Biacore AB (Sweden). The production of mouse anti-human TC mAbs [13], human TC [14], and the synthesis of biotin-Cbl conjugate [15] have been described previously. The phagmid-displayed random peptide libraries (two linear 14-mer and one disulfide constrained 9-mer peptide library) were from Cosmix GmbH, Germany.

### SPR studies

Surface plasmon resonance measures in real time the changes in mass bound to a sensor chip. It detects the changes in refractive index of the surface layer of a solution in contact with the sensor chip that are caused by a variation of the mass on the sensor chip surface. SPR binding was performed using a Biacore instrument (Biacore AB) according to the recommendations of the manufacturer.

#### Protocol 1

Rabbit anti-(mouse Fc-γ) IgG (30 mg /L) was immobilized on the surface of the carbodiimide-activated sensor chip. The reaction was performed in 50 mmol/L acetate buffer, pH 5.0 at a flow rate of 5 μL/min, until the SPR signal reached ~2000 resonance units over baseline. Unreacted groups were blocked using 1 mol/L ethanolamine, and the primary antibody, mouse anti-(human TC) IgG, 10 mg/L, was captured on the sensor chip in Hepes-buffered saline, pH 7.3, 3.4 mmol/L EDTA, 50 mg/L SPR surfactant (HBS-EP). Mouse serum (1:10 dilution) was then injected in order to saturate the excessive binding sites on the anti-(mouse epitope) IgG sensor chip.

Interaction of recombinant apo- or holoTC with the antibodies on the sensor chip was evaluated at TC = 1 nmol/L to 10 μmol/L, flow 5 μL/min. The Ag/mAb complex was dissociated by washing with 10 mmol/L HCl after each analysis. The covalently immobilized rabbit anti-(mouse Fc-γ) IgG was stable and there was no significant decrease in the ligand binding during repeated washing and reuse of the sensor chip. Data points were collected, and the rate constants for association and dissociation (k_on _and k_off_) were calculated. The equilibrium dissociation corresponded to K_D _= k_off_/k_on_. In some experiments antibody 3–11 was substituted for the rabbit anti-(mouse Fc-γ) IgG as primary immobilized antibody.

In experiments with binding of phagemid-displayed peptides to antibodies, the phagemids were diluted in HBS-EP to 1.6 10^12 ^cfu per mL, equaling a peptide concentration of about 3 nmol/L, and reacted with the immobilized antibody at a flow rate of 10 μL/min. After each run the sensor chip was regenerated with 200 mmol/L glycine, pH 2.2. In competition experiments between holoTC and phage-displayed peptides for binding to mAb 3C4, holoTC was used at 1 μmol/L.

#### Protocol 2

Biotin-Cbl (100 μmol/L) was immobilized on a streptavidin (SA) sensor chip. The immobilized Cbl was saturated with apoTC (1 μmol/L) forming bound holoTC and mAb 3C4 diluted in phosphate buffer of different pH and ionic strength was injected. The proteins were stripped from the SA sensor chip with 200 mmol/L glycine, pH 2.2 prior to reuse.

### Panning of phagemid-displayed random peptide libraries with mAb 3C4

MAb 3C4 (40 g/L) was immobilized on paramagnetic beads (10 g/L). The reaction was performed in 10 mmol/L acetate buffer, pH 4.0 for 45 min at ambient temperature. Unreacted groups were blocked using 1 mol/L ethanolamine. After washing, 20 μL of beads were mixed with about 10^12 ^packaged phagemids from each library and incubated for 1 h. Uncoated carboxybeads were used as a control. After incubation, beads were washed with PBS containing 0.05% Tween 20. Bound phagemids were eluted with 200 mmol/L glycine buffer, pH 2.2 and immediately neutralized with 1 mol/L Tris, pH 8.0. Amplification and purification and selection of specific phagemid peptides was performed using standard protocols. In brief, ELISA wells were coated with mAb 3C4 (100 ng/well) or unrelated antibody over night at 4°C and then blocked with 10 mg/mL BSA in PBS (PBA) for 1 h at RT. Centrifuged packaged phagemids were added at a concentration of 10^10 ^to 10^8 ^cfu per well in PBS and incubated for 1 h. Wells were washed 5 times with PBS containing 0.1% Tween-20 and 3 times with PBS. Bound phage particles were detected using HRP-conjugated anti-M13-antibody in PBS.

### Binding of ^57^Co-cobalamin labeled TC to anti-human TC monoclonal antibodies

Monoclonal anti-TC IgG were bound to polyclonal goat anti-(mouse epitope) IgG that were covalently linked to magnetic microspheres as described previously [11]. TC in 1.8 mL of human serum was labeled with ^57^Cbl (300 pmol/L, 30 min). A 90 μL aliquot of the radiolabeled serum was incubated with 10 μL of each anti-TC IgG coated microsphere preparation at room temperature for 1 h, the microspheres were separated using a magnet, and the radioactivity in each fraction was determined. An equivalent amount of ^57^Cbl in PBA was used to determine non-specific binding.

To assess the binding of native apoTC, a 90 μL aliquot of unlabeled serum was incubated with 10 μL of each anti-TC IgG coated microsphere preparation at room temperature for 1 h. Microspheres and supernatant were separated using a magnet. ApoTC bound to the microspheres was labeled with ^57^Cbl in 100 μL PBA (300 pmol/L, 30 min), separated using a magnet, and the radioactivity measured. ApoTC remaining in the supernatant was determined by labeling with ^57^Cbl (300 pmol/L, 30 min) and incubating for 1 h with 15 μL mAb 3–9 coated microspheres, sufficient to bind all TC in the sample. Microspheres were separated using a magnet and the bound radioactivity determined.

### Conjugation of antibody 3–11 with HRP

Monoclonal antibody 3–11 was coupled to horse-radish peroxidase (HRP) using Ez-link™ Maleimide Activated HRP kit (Pierce, USA) and a protocol supplied by the manufacturer. In short, mAb 3–11 in PBS was modified with a 25-fold molar excess of N-succinimidyl S-acetylthioacetate in dimethylformamide and after 30 min, deprotected with a 90-fold molar excess of hydroxylamine for 2 h at ambient temperaure. The thiolated antibody was desalted and then reacted with an eight-fold excess of EZ-Link™ Maleimide Activated HRP at ambient temperature for 1 h and dialyzed three times against 100 volumes of PBS.

### Non-isotopic sandwich assay for holoTC using mAb 3C4 as capture antibody

Maxisorb plates (NUNC-Immuno™, Denmark) were coated with 10 μg/mL mAb 3C4 in PBS over night at 4°C and then blocked with PBA for 2 h at ambient temperature. Serum (50 μL) and PBS (50 μL) were added to each well and incubated at ambient temperature for 30 min. After washing three times with 200 μL of PBA containing 0.05% Tween 20 (wash buffer), 100 μL of HRP-conjugated mAb 3–11 was added and the mixture was incubated for 30 min at ambient temperature. Each well was washed three times in wash buffer and 100 μL of the HRP-substrate, TMB (BioFx inc., USA) was added and incubated for 10 min. The reaction was quenched with 100 μL 0.12 mol/L HCl and the color developed was measured at a wavelength of 450 nm.

To produce a calibration curve, 10 mL of serum was treated with an excess of the high affinity mAb 3–9 immobilized onto magnetic microspheres, so as to remove all TC in the sample. Another 10 mL of the same serum was saturated with cyanoCbl. The individual calibrators were then constructed by mixing the two serum pools in different ratios, giving values from 390 to 7 pmol/L of holoTC. HoloTC values were determined using the commercially available holoTC assay kit HoloTC RIA (Axis-Shield, Norway).

## Results

### SPR analysis

**Figure 1:**
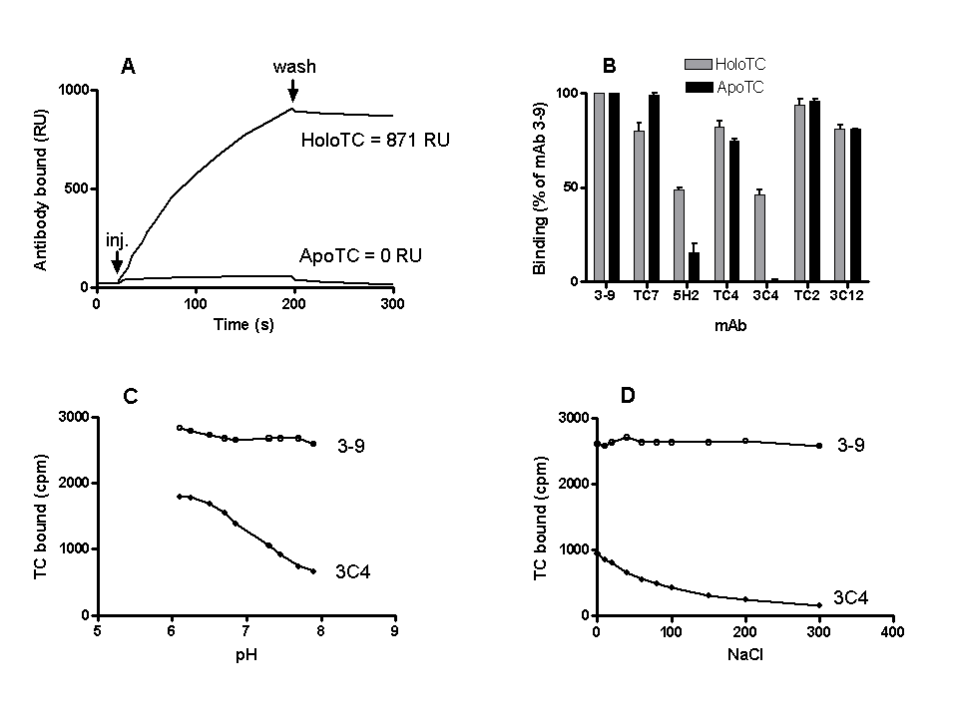
**Binding of holoTC and apoTC by anti-human TC mAbs**. (A) SPR sensorgram of the binding of mAb 3C4 to immobilized recombinant holoTC and apoTC. HoloTC and apoTC were first bound to mAb 3–11, which was covalently attached to the sensor chip, and then mAb 3C4 was injected over the sensor chip. (B) The affinity and specificity of seven different antibodies for native holoTC and apoTC. The Figure depicts one experiment using quadruplicate assays. Antibodies were immobilized on paramagnetic microspheres. See Material and Methods. (C) Binding of ^57^Cbl holoTC to mAb 3C4 and mAb 3–9 as a function of pH. (D) Binding of ^57^Cbl holoTC to mAb 3C4 and mAb 3–9 as a function of ionic strength at pH 7.3.

The binding properties and epitope specificity of 9 mAbs were determined by SPR measurements in a Biacore instrument. Recombinant apoTC or holoTC was immobilized on the chip via the first mAb attached to rabbit anti-(mouse epitope) IgG, whereupon the second mAb was injected. All mAbs were combined in all possible permutations. This analysis identified one antibody, 3C4 that did not bind apoTC (Fig. 1A). The specificity of mAb 3C4 for holoTC was at least 100-fold higher than for apoTC (Table 1). From the sensorgams a K_D _= 75 nmol/L was determined for the binding of mAb 3C4 to holoTC. Two other antibodies, 5H2 and 4–7 (epitope cluster 2) exhibited weak preference for holoTC over apoTC (Table 1). The remaining mAbs recognized different epitopes but reacted equally with both apoTC and holoTC. Our previous studies [15] had indicated that mAbs TC2 and 3C4 bound to the same epitope cluster 4 because binding of one mAb completely blocked the binding of the other mAb. However, the current experiments showed a clear difference in the ability of the two mAbs to interact with apoTC and holoTC. Therefore, mAb 3C4 has been reassigned to a new epitope 6. The specificity of mAb 3C4 for holoTC suggested that the bound Cbl may be an integral part of the corresponding epitope. To investigate this, mAb 3C4 was injected over a streptavidin sensor chip coated with biotin-Cbl [15]. No binding of mAb 3C4 directly to the immobilized Cbl was observed. Likewise, a 15-fold molar excess of cyanoCbl did not inhibit the binding of mAb 3C4 to immobilized holoTC.

**Table 1:** Binding kinetics for anti-human TC mAbs as measured by SPR

**MAb**	**Isotype**	**Epitope**^1^	**K_D_**^2 ^(nmol/L)	**Holo/apo specificity **(-fold)
3–9	IgG1	1	0.03	1
3–11	IgG2a	2a	0.2	1
TC7	IgG2a	2a	10	1
4–7	IgG2b	2b	0.5	3
5H2	IgG1	2b	>100	4
TC4	IgG1	3	5–10	1
TC2	IgG1	4	1	1
3C12	IgG1	5	5	1
3C4	IgG1	6	none	>100

^1 ^Epitope nomenclature follows that given in ref. 15. Mab 3C4 was originally assigned the same epitope as TC2 (epitope 4). Based on additional data, it has been reassigned a new epitope 6.

^2 ^Approximate K_D _values for apoTC.

### Evaluation of holoTC specificity for mAb 3C4 immobilized on magnetic microspheres

The specificity of mAb 3C4 was further investigated using native TC in serum. To determine binding of apoTC, mAb-coated microspheres were incubated with untreated serum, and to determine binding of holoTC they were incubated with serum containing ^57^Cbl-labeled holoTC (see Experimental Procedures). The results corroborated those seen in the SPR experiments (Fig. 1A, 1B). MAb 3C4 bound holoTC but showed no significant binding of apoTC. The specificity of 3C4 for holoTC was at least 70-fold higher than for apoTC.

### Factors influencing the binding characteristics

The binding of ^57^Cbl labeled holoTC by mAb 3C4 immobilized on magnetic beads was affected by pH and ionic strength. Binding increased at lower pH, reaching a plateau at about pH 6.1, the pI for TC (Fig. 1C), and decreased with increasing ionic strength. (Fig 1D). Altering pH or ionic strength did not affect the specificity, and apoTC was not bound above background level at any pH or ionic strength tested. Other anti-human TC antibodies (mAbs 3–11 and 3–9) were not significantly affected by either variable. There was no significant difference between different anions (Cl, SCN, SO_4_) or cations (Na, K, Mg, Ca) (data not shown). SPR data indicated that although both on and off-rates were affected by pH and ionic strength the effect on the on-rate was predominant when holoTC bound immobilized mAb 3C4 (Table 2). When holoTC was immobilized on the sensor chip and mAb 3C4 was eluted over it, pH and ionic strength effects largely disappeared. Presence or absence of 3 mM EDTA had no effect.

**Table 2:** Dependence of binding kinetics on pH and ionic strength as measured by SPR

**Immobilized reagent**	**pH**	**NaCl **(mmol/L)	**k_on _**(10^4 ^M^-1^s^-1^)	**k_off _**(10^-4 ^s^-1^)	**K_D _**(nmol/L)
HoloTC	6.0	0	10	1	1
	7.4	0	8	0.4	0.5
	7.4	300	7	0.6	0.9
	8.0	0	7	0.2	0.3
MAb 3C4	6.0	0	50	4	0.8
	6.0	300	0.3	35	1340
	7.4	0	3	14	46
	7.4	300	0.3	41	1380
	8.0	0	7	19	27

### Epitope mapping of mAb 3C4 using phage-displayed peptide libraries

**Figure 2:**
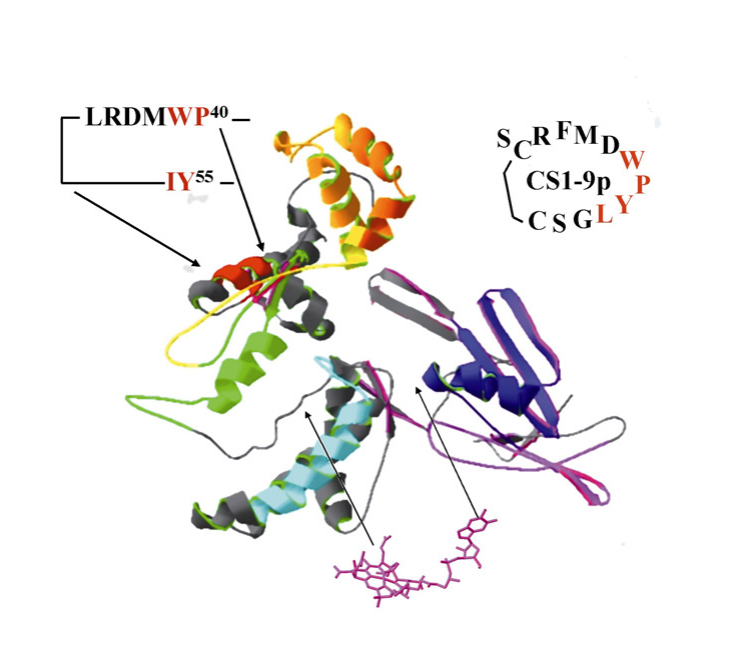
**The mAb 3C4 phagotope CS1-9p and the location of the corresponding putative epitope P40 – Y55 in the simulated three-dimensional model of TC. **(epitope 6, red). The epitope clusters identified in [15] are also shown. Epitope clusters 1 (pink), 2 (orange), 3 (magenta), 4 (green), 5 (yellow), and heparin-binding site (blue). The putative cobalamin-binding region is also indicated.

Previous results had shown that mAb 3C4 bound only native holoTC, whereas neither reduced TC nor fragments of TC were recognized by mAb 3C4 [15]. This observation together with the failure of mAb 3C4 to bind apoTC or Cbl indicated that the epitope of mAb 3C4 is discontinuous with the relevant residues dissipated along the amino acid sequence and brought into close proximity only after binding of Cbl. To elucidate this further, we used phage display to map mAb 3C4, as this technique is often successful in identifying both continuous and discontinuous epitopes [16]. Three phage-displayed random peptide libraries were screened with mAb 3C4, two linear 14-mer libraries and one disulfide constrained 9-mer library. The mAb did not react with any specific peptides in the two linear peptide libraries, suggesting the absence of any continuous epitope. In contrast, the constrained peptide library, which has a greater probability of identifying discontinuous epitopes, generated a series of specific phagotopes forming two similar motifs (Table 3). These phagotopes could not be aligned to any specific sequence in human TC, which is in agreement with a discontinuous epitope. A comparison of the phagotopes with an in silico generated model of TC [15] identified two structures in the N-terminal part of TC that resembled the motif. Both structures have the main sequence in common. One structure is composed of the anti-parallel sequences P^40^WMDRL^45 ^+ I^54^Y^55 ^and is a predicted solvent exposed loop (Fig. 2). The other structure is composed of the parallel sequences P^40^WMDRL^45 ^+ Y^72 ^LHS^75^. The binding motif therefore represents a mimotope of the true epitope on holoTC.

**Table 3:** Phagotope sequences specific for mAb 3C4. Phagotopes were identified from a phage-displayed disulfide constrained 9-mer random peptide library.

Motif 1	CS-x1-x2-**Y**-x3-**W**-D-x4-x5-x6-CS
	x1 = F, G, L
	x2 = F, L, R
	x3 = L, P, Q
	x4 = M, Q, Y
	x5 = D, F
	x6 = M, R
Motif 2	CS-F-F-Y-S-L-C-**Y**-C-**W**-CS

The phagemid displayed peptides were specific for mAb 3C4 (Table 4). Phagemids CS1-6p, CS1-11p, and CS1-31p were investigated for binding to five other anti-human TC mAbs (3–9, 5H2, TC4, TC2, and 3C12) that recognized five different epitopes on TC. Whereas the three phagemid-displayed peptides bound to immobilized mAb 3C4 with 24, 32, and 42 RU, respectively, no binding was observed to the other five mAb (0 RU). The SPR signals were rather low, but phages do not cause strong SPR signals for reasons that are thought to depend on the large size of the phage itself [17]. The interaction between phagemids and mAb 3C4 was inhibited in a dose dependent manner by holoTC with IC_50 _values between 0.2 and 0.4 μmol/L (Table 4). These values are in good agreement with the K_D _values estimated from the sensorgrams, which were 75 nmol/L for the binding of holoTC to mAb 3C4 and about 1 nmol/L for the binding of mAb 3C4 to the different phagotopes. According to the Cheng-Prusoff equation, IC_50 _= K_I _(1 + S/K_D_) [18] and assuming that for holoTC K_D _≈ K_I_, holoTC would inhibit 3 nmol/L of phagotope with an IC_50 _≈ 0.3 μmol/L. Although the peptides when bound to the phagemid were specific, chemically synthesized peptides did not inhibit the binding of 3C4 to either holoTC or the same peptides bound to phagemid, demonstrating the importance of mimotope presentation for its activity.

**Table 4:** Binding of phagemids to mAb 3C4 and blocking by holoTC.

**Phagemid**	**No. of clones**	**Sequence**	**Phage bound **(RU)	**HoloTC IC50 **(μM)
CS1-2p	1	csLFYLWDQDRcs	4	-
CS1-6p	10	csFRYLWDQDRcs	24	0.4
CS1-9p	10	csGLYPWDMFRcs	35	0.3
CS1-11p	10	csFRYQWDMFRcs	32	0.2
CS1-29p	1	csFFYSLCYCWcs	20	0.3
CS1-31p	2	csGLYPWDYFMcs	42	0.3
CS1-37p	1	csARYSWDRLRcs	0	-

### Non-isotopic sandwich assays for holoTC

**Figure 3:**
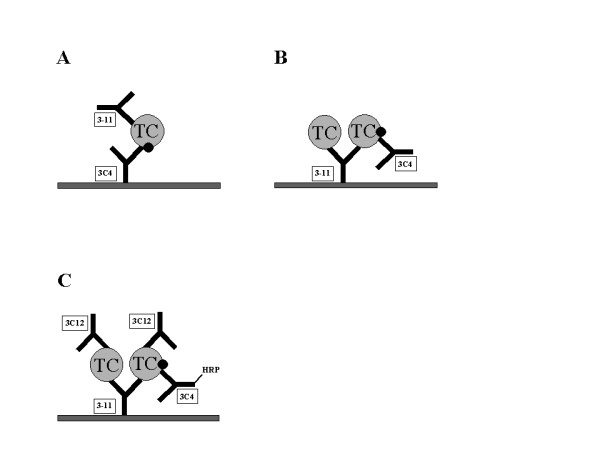
**Three different assay formats for the direct measurement of holoTC. **(see main text for details). (A) MAb 3C4 as capture ligand for holoTC and mAb 3–11 as detecting ligand. (B) MAb 3–11 as capture ligand for total TC and mAb 3C4 as detecting ligand for holoTC. (C) MAb 3–11 as capture ligand for total TC and mAb 3C4 as detecting ligand for holoTC and mAb 3C12 as detecting ligand for total TC.

Three different assay formats were investigated, using mAb 3C4 as the capture or the detecting ligand, and measurement of holoTC or the Cbl saturation ratio of TC. (Fig. 3).

#### MAb 3C4 as capture ligand

**Figure 4:**
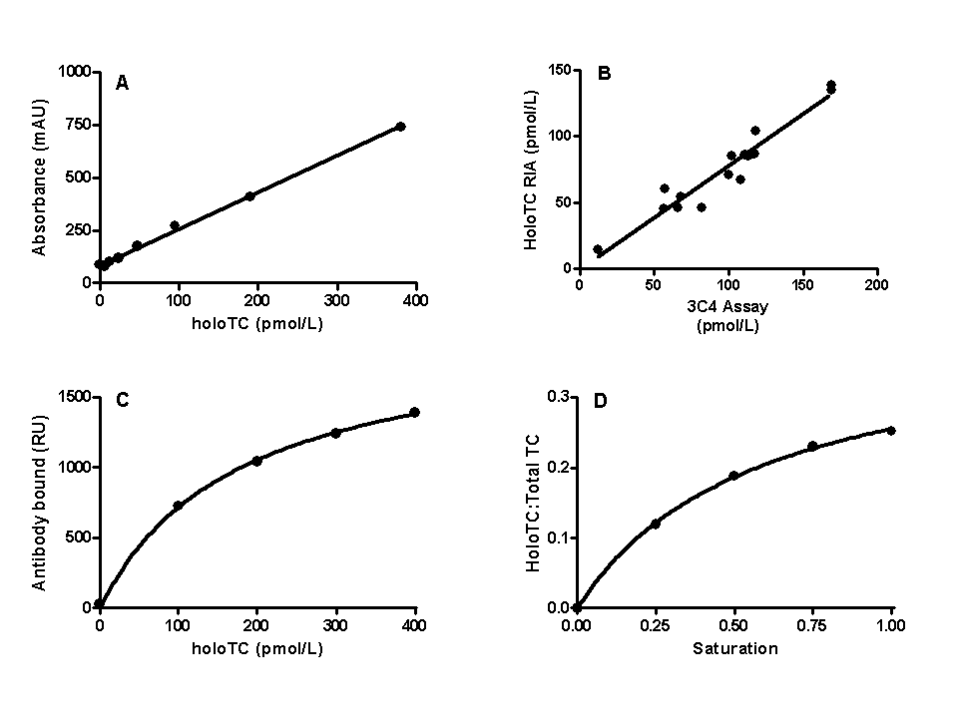
**Calibration curves for the three assay formats of Fig. 3, and a comparison of prototype assay with HoloTC RIA. **(A) Calibration curve for prototype assay using mAb 3C4 as capture ligand (format A). (B) Comparison of the prototype assay and HoloTC RIA for 15 serum samples ranging from 14 to 139 pmol/L as determined by HoloTC RIA. (C) Calibration curve for assay using mAb 3C4 as the detecting ligand (format B). (D) Calibration curve of assay for cobalamin TC saturation (format C).

The assay protocol is described in Experimental Procedures. Different concentrations of recombinant human holoTC were added to wells coated with mAb 3C4 and bound holoTC was detected using mAb 3–11 conjugated to HRP (Fig. 3A). A typical calibration curve is depicted in Fig. 4A. Fifteen serum samples with holoTC values ranging from 14 to 139 pmol/L, as determined by HoloTC RIA [11], were quantified by 3C4 capture assay. A comparison of the two methods showed good correlation; Y = 0.78X-0.40, r = 0.96 (Fig 4B).

#### MAb 3C4 as detecting ligand

This was investigated in the Biacore. Mixtures of apoTC and holoTC were captured on immobilized mAb 3–11. The amount of holoTC bound was detected by a fixed concentration of mAb 3C4 (Fig. 3B). The calibration curve obtained fitted a one-phase binding hyperbole, Y = 1993X/(178-X); r^2 ^= 1.00 (Fig. 4C). As the serum level of holoTC is too low for measurement in the Biacore, no comparison could be made with the HoloTC RIA method.

#### Measurement of the cobalamin saturation ratio of TC

It has been suggested that the cobalamin saturation ratio of TC (holoTC:total TC) provides a better index of vitamin B12 deficiency than the absolute holoTC values [9]. Therefore we investigated such a format in the Biacore. Different mixtures of apoTC and holoTC were captured on immobilized mAb 3–11. The amount of holoTC bound was detected by a fixed concentration of mAb 3C4 and the total amount of TC by mAb 3C12 (Fig. 3C). As mAb 3C4 is specific for holoTC whereas mAb 3C12 does not discriminate between the holo and the apo-form, and the two antibodies do not overlap (15), the saturation ratio could be determined. The calibration curve obtained fitted a one-phase binding hyperbole, Y = 0.40X/(0.58-X); r^2 ^= 1.00 (Fig. 4D).

## Discussion

We have previously determined the epitope specificity of a panel of 17 monoclonal antibodies and their relation to Cbl and receptor binding regions in TC [15]. In the present work our aim was to identify epitopes that are unique to the holo protein, with the over-all objective to develop an assay for the direct determination of holoTC in plasma. Since this fraction of TC represents newly absorbed and circulating Cbl available for cellular uptake, the quantitative estimation of this fraction may provide a more accurate measure of physiological Cbl status than the currently used total serum Cbl status.

The 9 mAbs used in the present work represented all 5 epitope clusters previously identified using holoTC [15]. We obtained an identical epitope map using apoTC, with one exception, mAb 3C4 did not bind to apoTC. MAb 3C4 was highly specific for holoTC, and no binding of apoTC above the background level was observed in either SPR experiments or with labeled serum and antibodies immobilized onto magnetic beads. Such high specificity for the holo-form may be achieved by an epitope that either encompasses the cobalamin moiety or is induced by the binding of cobalamin, i.e. cryptic continuous or discontinuous epitopes. However, the inability of mAb 3C4 to bind to immobilized cobalamin and of cobalamin to interfere with the binding of mAb 3C4 to immobilized holoTC make it highly unlikely that Cbl is a part of the epitope. Continuous or linear epitopes contain 4–8 amino acids next to each other in the primary sequence and are usually recognized by the antibody in both native and denatured protein. The presence of a continuous epitope in holoTC was considered unlikely for the following reasons: mAb 3C4 did not bind to (i) apoTC, which, apart from lack of Cbl, is only conformationally different from holoTC, (ii) reduced TC or (iii) fragments of TC. Discontinuous epitopes, which consist of amino acids separated in the primary sequence but brought together in the native protein, may be successfully mapped by using phage-displayed random peptide libraries [16]. We screened three peptide libraries, two linear 14-mer libraries and one disulfide constrained 9-mer library. Whereas the two former yielded no specific sequences, a finding in agreement with a discontinuous epitope for 3C4, the constrained library generated a series of specific phagotopes forming one or possibly two similar motifs. The phagotopes were true mimics of the epitope since they did not bind other TC specific antibodies and the binding to mAb 3C4 was inhibited by holoTC. Such mimics of epitopes, mimotopes may or may not show similarities with the epitope at the amino acid level. We have, as previously reported [15], generated a computer based three-dimensional model of apoTC using the de novo modeling procedures on the automated protein modeling server HMMSTR/Rosetta [19]. In the absence of any homologous structures, the accuracy of the model cannot be validated, and we have used it for visualization purposes. However, these models often have a higher degree of accuracy at the local structural level [20], and this applies in particular to the Rosetta method, which ranks as the best 3D predicting program available [21]. Therefore, we investigated the model for structures homologous to the identified motif. As the model is of apoTC to which mAb 3C4 does not bind we would not expect to find any striking similarity. However, based on the sequences of the reactive phage inserts two epitope motifs in the N-terminal part of TC could be inferred from the molecular model of apoTC. The two epitope motifs have their main sequence, P^40^W^41^M^42^D^43^R^44^, in common. This is associated with either I^54^Y^55^, which is localized in the same predicted solvent-exposed loop as the main sequence, or with Y^72^L^73^H^74^S^75^, which is part of an adjacent parallel alpha-helix. Both proposed motifs (PWMDR + IY or PWMDR + YLHS) are localized on the opposite side of TC relative to epitope clusters 1, 2, and 3. This is in agreement with previous results indicating that the epitope of mAb TC2 (epitope 4), which is mutually exclusive with 3C4 on holoTC, is on the opposite side of the TC molecule compared to the epitope clusters 1, 2, and 3 [15]. The epitopes of antibodies TC2 and 3C4 are not in close proximity in the primary sequence. However, it is known that TC and the closely related Cbl-transporting protein, intrinsic factor, change their conformation when binding Cbl, which results in a reduced Stokes radius [22]. In addition, it was recently demonstrated that Cbl assembles distant domains of intrinsic factor in a more compact structure with high affinity for Cbl and its specific receptor [23,24]. It seems reasonable to assume that a similar transformation occurs in TC in view of the 28-fold higher affinity of holoTC for the receptor compared to apoTC [5]. In line with this assumption, the epitopes of mAb TC2 (epitope 4) and mAb 3C4 (epitope 6) are quite close in the 3D model of TC (Fig. 4). We conclude that mAb 3C4 binds to a discontinuous epitope existing only in holoTC and tentatively localize it to the loop P^40^W^41^M^42^D^43^R^44^-X_9_-I^54^Y^55 ^or the parallel alpha-helixes P^40^W^41^M^42^D^43^R^44 ^and Y^72^L^73^H^74^S^75 ^in holoTC (Fig. 2). MAb 3C4 has been previously assigned to epitope cluster 4 because it is mutually exclusive with mAb TC2 on holoTC [15]. Based on the new finding that mAb 3C4 does not recognize apoTC, it has been reassigned to the new epitope 6. Likewise, mabs 5H2 and 4–7, but not mAbs TC7 and 3–11, all binding to epitope cluster 2, display preferential binding to holoTC over apoTC (Table 1). Therefore epitope cluster 2 has been subdivided into 2A and 2B. Fig. 2 shows all six epitope clusters and the putative binding site for Cbl inferred from the findings that (i) the conserved region S^192 ^– L^206 ^is important for Cbl binding [25], (ii) the C-terminal domain A^320 ^– W^427 ^is involved in Cbl binding, albeit weakly [15], and (iii) mAb 3–9 (epitope cluster 1) suppresses Cbl binding to TC [13]. As indicated in Fig. 2, the holoTC specific region is thus localized far away from the Cbl-binding region.

The affinities of the antibody-antigen reaction varied inversely with ionic strength. In particular, the association rate constant was negatively affected by salt concentration, suggesting that electrostatic forces contributed to antigen binding. Also pH affected the interaction with affinity increasing with decreasing pH. However, the sequences of the phagotopes do not support a dominant role for ionic interactions in the epitope-paratope reaction. It seems more likely that these effects are due to additional interactions away from the epitope-paratope region. Interestingly, when mAb 3C4 was allowed to bind to immobilized holoTC, the pH and ionic strength effects largely disappeared, neither k_on _nor k_off _were significantly affected. With respect to k_off_, this may be explained by the difference in valency of binding. HoloTC is bound monovalently to mAb3C4 whereas mAb3C4 is bound bivalently to holoTC. MAb3C4 is thus more stably bound and will be released at a lower rate. However, it is possible that immobilized holoTC can become more refractory to a conformational change induced by pH and ionic strength alterations. An antibody to a discontinuous epitope would be more susceptible to changes in the antigen presentation than an antibody with a continuous epitope, such as mAbs 3–11 and 3–9.

The main objective of this study was to investigate the possibility to develop direct assays for holoTC that are adaptable to the larger clinical instruments. The existing commercially available assay for holoTC, HoloTC RIA, is a manual radioassay, which is based on the principle that all TC in a sample is captured and separated whereupon Cbl is released and measured in a competitive radioassay [11]. Although being robust and sensitive, it does not lend itself easily to automation, and thus cannot be used to measure the many hundreds to thousands of samples sent to the clinical laboratory. In addition it requires 400 μL of sample. The identification of a mAb with a high specificity for holoTC has provided a unique tool to develop an assay that is specific, direct, and can be easily automated. We here give proof of concept for three different assay formats; one using mAb 3C4 as the capture reagent, one as the detecting reagent and one measuring the saturation ratio (Fig. 3). The saturation ratio (holoTC:total TC) may be a better representative of cobalamin concentration [9] and has the advantage of being independent of sample volume. Because of the dependency of the antigen-antibody interaction on ionic strength when the antigen is in the free phase, as discussed above, an assay format using the holoTC specific antibody as the detecting reagent would be best suited for further development. However, because of non-specific binding of the holoTC specific antibody to the elisa plate surface, the signal-to-noise ratio was superior with a format using the holoTC specific antibody as the capture reagent. Therefore we chose to develop the first format and compare this to the commercial assay using 15 different serum samples. The correlation was good with Y = 0.78X-0.40 and r = 0.96 (Fig. 4B). The slope differed from unity, but that may be explained by the use of different calibrators in the two assays. In HoloTC RIA was used the calibrators supplied with the kit that are made up of recombinant holoTC. In the prototype assay we used serum-based calibrators, which as yet have not been normalized against the recombinant calibrators.

## Conclusion

We have identified a monoclonal antibody with a unique specificity for the holo-form of human transcobalamin. The specificity arises from conformational changes occurring in transcobalamin upon binding of cobalamin and cobalamin as such is not part of the epitope. Using this antibody we have successfully developed a prototype assay that allows the simple and direct quantification of holoTC in human blood. We are currently continuing the development of this prototype assay into an automatic holoTC test for routine use.

## Abbreviations

TC, transcobalamin; holoTC, holotranscobalamin; apoTC, apotranscobalamin; HC, haptocorrin; Cbl, cobalamin; SPR, surface plasmon resonance, RU, resonance unit.

## Competing interests

L. Orning, A. Rian, A. Campbell, J. Brady are employees of Axis-Shield ASA, which has the proprietary rights to the antibody described. The departments of E.V. Quadros and S. Fedosov have license agreements with Axis-Shield regarding supply of reagents used in the assay HoloTC RIA. The authors do not consider that these agreements cause any conflict of interest because the submitted manuscript only uses HoloTC RIA for purposes of comparison.

## Authors' contributions

LO: main writer and study concept – AR, AC, JB: assay development – SF, KT: antibody work – BB: phage display -EVQ: antibody work and study concept.
